# *Smad7* Is Highly Expressed in Human Degenerative Discs and Participates in IL-1*β*-Induced Apoptosis of Rat AF Cells via the Mitochondria Pathway

**DOI:** 10.1155/2022/2912276

**Published:** 2022-06-26

**Authors:** Bo Li, Ze-Yu Lu, Sheng-Dan Jiang, Lei-Sheng Jiang, Xin-Feng Zheng

**Affiliations:** Spine Center, Xinhua Hospital, Shanghai Jiaotong University School of Medicine, Shanghai 200092, China

## Abstract

**Background:**

Abnormal *Smad7* expression can lead to apoptosis in different cell types. Previously, we found high expression of *Smad7* in rat degenerative discs. However, the exact role of *Smad7* in the apoptosis of disc cells and the possible underlying mechanism remain unclear.

**Methods:**

Degenerative and nondegenerative human lumbar intervertebral discs were collected from patients during operation. The expressions of *SMAD7* mRNA and protein in the different components of these discs were measured with real-time PCR and Western blotting, respectively. Annulus fibrosus (AF) cells were isolated and cultivated from the discs of young healthy rats. *Smad7* in the AF cells was overexpressed with adenovirus and knocked down with siRNA. *IL-1β* was used to induce apoptosis in the AF cells. Loss-and-gain cell function experiments were performed to show the effect of *Smad7* on the apoptosis of AF cells. The function recovery experiments were performed to verify whether *Smad7* regulates the apoptosis of AF cells through the mitochondria-mediated pathway.

**Results:**

Both the mRNA and protein expressions of *Smad7* were significantly higher in the different components of human degenerative discs than in those of the nondegenerative discs. *IL-1β* stimulated apoptosis while upregulating the *Smad7* expression in the AF cells *in vitro*. Overexpression of *Smad7* in AF cells exaggerated the *IL-1β*-induced apoptosis in the cells while knockdown of *Smad7* expression suppressed this apoptosis. With the exaggerated apoptosis in the AF cells with *Smad7* overexpression, both active cleaved caspase-3 and cleaved caspase-9, the ratio of Bax/Bcl-2, and Cyt-c increased significantly. However, the inhibitor of caspase-9, Z-LEHD-FMK, significantly diminished the apoptosis in these cells.

**Conclusion:**

*Smad7* is highly expressed in human degenerative discs and participates in *IL-1β-induced apoptosis* of rat AF cells via the mitochondria pathway. *Smad7* may be a potential target for the prevention and treatment of degenerative disc disease.

## 1. Introduction

Intervertebral disc degeneration disease (IVDD) is one of the most common causes of disability in aging people and a decline in the quality of their life [1–4]. However, the pathogenesis of IVDD is not fully understood. A decrease in the number of live cells in the disc and reduced intracellular matrix with altered components are the pathological basis of disc degeneration [2, 5]. Increased apoptosis of disc cells is the primary reason for the decrease in the number of live cells [2, 5, 6]. Excessive apoptosis could also lead to a decrease in cell density and an increase in intracellular matrix catabolism, resulting in disc degeneration [7–9]. Thus, suppressing the excessive apoptosis of disc cells is believed to be a potential way to delay disc degeneration.

*Smad7* is a key member closely related to *TGF-β* functions. It binds to the *TGF-β* receptor, inhibits the phosphorylation of Receptor-Smad complex, and blocks the signal transduction of *TGF-β*. Meanwhile, *TGF-β* could induce *Smad7* expression through the activation of Receptor-Smads, thus forming a positive feedback regulation. Activated Receptor-Smads could also upregulate *Smad7* expression through the activation of *Smad7* promoter, forming another regulatory feedback loop [10]. By blocking *TGF-β* signaling pathways, *Smad7* inhibited the synthesis of collagen and proteoglycans. It has been reported that adenovirus with *Smad7* cDNA transfected into hepatic stellate cells or renal interstitial fibroblasts inhibited the synthesis of collagen and proteoglycan and stopped the process of fibrosis [11, 12]. Our previous study also has demonstrated that *Smad7* plays key roles in disc degeneration. *Smad7* was found to be highly expressed in degenerated intervertebral discs in rats, and *Smad7* expression was closely associated with the metabolism of the intracellular matrix [13]. It has been reported that *Smad7* promoted apoptosis by blocking the NF-*κ*B/p65 pathway in glomerular podocytes [14]. However, the exact role of *Smad7* in the apoptosis of disc cells and the possible underlying mechanism remain unclear.

*IL-1β* is significantly increased in degenerative intervertebral discs (IVDs) [15]. An increasing number of studies have proved that *IL-1β* is involved in the inflammatory response in the process of intervertebral disc degeneration. Exposure of human AF cells to *IL-1β* can significantly increase apoptosis [16, 17]. Our previous study also confirmed that *IL-1β* is involved in IVDD and *IL-1β* enhances the effect of serum deprivation on rat AF cell apoptosis [18]. *IL-1β* was reported to induce the expression of *Smad7* in chondrocyte [19]. But it is still unclear whether *Smad7* participates in *IL-1β*-induced AF cell apoptosis.

Based on the above-mentioned findings, we continue our study on the role of *Smad7* in disc degeneration. In this study, we further investigated the expression of *SMAD7* in human degenerative discs and clarified the role of *Smad7* in the *IL-1β-induced apoptosis* of rat AF cells. The underlying signaling pathways of apoptosis are also explored. We hope our results will provide a new idea for the prevention and treatment of IVDD.

## 2. Materials and Methods

### 2.1. Human Disc Sample Collection

Human disc tissues were collected from patients who underwent operations for lumbar spine diseases. A total of 45 segments of intervertebral disc tissue were collected. According to Pfirrmann's grading criteria on MRI, 4 discs were grade I, 10 were grade II, 11 were grade III, 11 were grade IV, and 9 were grade V. These 45 discs were divided into 2 groups: nondegenerative (grades I-II) and degenerative (grades III-V). The annulus fibrosus, nucleus pulposus, and endplate tissues of each disc were carefully separated in the operation table and appropriately stored for the following experiments. Human AF tissue collection and animal experiments were approved by the Ethics Committee of our university hospital (No. XHECD2015107). All experiments involving human specimens followed the Helsinki declaration.

### 2.2. Isolation and Culture of Rat AF Cells

The whole lumbar spine segments were dissected from eight healthy 3-month-old male Sprague–Dawley rats after sacrifice by intraperitoneal injection of over-dosed Sumianxin. L1/2, L2/3, L3/4, L4/5, and L5/6 discs were separated. AF cells were isolated and cultured according to our previous method [18, 20]. First-passage cells maintained in a monolayer were used throughout the experiments [21]. The isolated primary AF cells were verified by the characteristics of their cell morphology [22].

### 2.3. Adenovirus-Mediated Gene Transfer into AF Cells

The adenovirus vector with *Smad7* gene overexpression was constructed by the manufacturer (Gene Pharma, China). On the day of transfection, AF cells were seeded into 24-well culture dishes at a density of 1 × 10^5^ cells/well. When they reached 50% confluence, cells were transfected with the *Smad7* overexpression adenovirus or negative control adenovirus at a multiplicity of infection of 100, and cells without transfection were used as control. The efficacies of transfection were determined by western blotting and real-time PCR.

### 2.4. Small Interfering RNA Transfection

*Smad7* small interfering RNAs were obtained from (Gene Pharma, China). si-*Smad7*-1, si-*Smad7*-2, and si-*Smad7*-3 sequences were listed as follows: si-*Smad7*-1: 5′-AGGCAUUCCUCGGAAGUCATT-3′; si-*Smad7*-2: 5′-GCAGCCTAACCAGACCTTT-3′; si-*Smad7*-3: 5′-GGCTGGAGGTCATCTTCAA-3′. Briefly, si-*Smad7* was added to OPTI medium (Gibco, USA) and then lipofectamine 3000 (Invitrogen, Carlsbad, California, CA) was added to OPTI medium (Gibco, USA) containing si-*Smad7*. After being incubated for 20 min, the mixtures were added to AF cells. Si-NC was served as a negative control. The efficacies of transfection were determined by western blotting and real-time PCR.

### 2.5. Quantitative Real-Time-PCR

Total RNA was obtained from AF cells using an RNA isolation kit (Invitrogen) according to the manufacturer's protocol. cDNA was generated by using the cDNA synthesis kit (Roche). A qRT-PCR method based on SYBR Green detection was performed for gene expression quantification using the primers. The primers were as follows: human *SMAD7* forward: 5′-CGGAAGTCAAGAGGCTGTGT3′, reverse: 5′-TGGACAGTCTGCAGTTGGTT-3′; Rat *Smad7* forward: 5′-AGGCATTCCTCGGAAGTCAA-3′, reverse: 5′-TGGACAGTCTGCAGTTGGTTTG-3′. Real-time-PCR was performed with ABI StepOne Plus equipment. GAPDH was used as an internal control to normalize the expression levels of different genes. The relative complementary DNA ratio was calculated using the value of threshold cycles (Ct). The amplification efficiency between the target and the reference control GAPDH was compared by using the 2^–*ΔΔ*Ct^ method.

### 2.6. Western-Blot Analysis

The AF cells were collected at 12, 24, and 48 h after recombinant adenovirus infection. The whole cell lysis was extracted using Cell Lysis Buffer for Western and IP (Beyotime, P0013) added with PMSF (1 : 100) [23]. The relative protein levels were calculated versus GAPDH. The primary antibodies used in this study were as follows: GAPDH, Bcl-2, Bax, Bcl-XL, Cyt-c, procaspase 9, cleaved-caspase 9, procaspase 3, cleaved-caspase 3, *Smad7* (Proteintech, USA, 1 : 1000), *Smad4* (Proteintech, USA, 1 : 1000), total-*smad2* (Proteintech, USA, 1 : 1000), total-*smad3* (Proteintech, USA, 1 : 1000), *P-smad2* (CST, USA, 1 : 1000), and *P-smad3* (CST, USA, 1 : 1000). Following incubation with a primary antibody, the membranes were washed with TBST and incubated with HRP-conjugated anti-rabbit or anti-goat second antibody. The membranes were then washed thrice, and the immunoreactive bands were visualized using NBT/BCIP as a substrate. Densitometric analysis was done using the NIH ImageJ Software to quantify the protein present in the detected bands. GAPDH content was assayed as standardization of sample loading. Quantitative densitometric values of each protein of interest were normalized to GAPDH.

### 2.7. Nuclear Staining Using Hoechst 33258

Three groups of AF cells were seeded in 96-well culture plates and added with Hoechst 33258 dye solution (Beyotime, C0003) followed by removing the culture media at 12, 24, and 48 h, respectively. The procedures were done as previously described [21]. After washing with PBS twice, AF cells were added with 0.5 ml 4% formaldehyde solution and fixed at RT for 10 min. Then, AF cells were washed with PBS for 2-4 times and incubated with Hoechst 33258 solution for 20-60 min in the dark at RT. Cells were observed under a fluorescence microscope under UV light followed by washing with PBS and adding with antifade reagent.

### 2.8. Cell Apoptosis Assay Using Flow Cytometry

AF cells overexpressing *Smad7* were first collected for apoptosis assay using flow cytometry at 12, 24, and 48 h, respectively. And then, we used LEHD-FMK to confirm the involvement of the mitochondria-mediated pathway in the apoptosis of rat AF cells after 48 h of *Smad7* overexpression. The experiments were performed according to the manual of Annexin V-FITC Apoptosis Detection Kit (BD Pharmingen TM) [7]. In brief, AF cells were collected by centrifuge at 1000 g for 5 min followed by wash with PBS and digestion using Trypsin-EDTA. After resuspension in PBS, cell numbers were counted and 5-10 × 10^4^ cells were aliquoted for staining with Annexin V-FITC solutions for 10 min in the dark at RT. Propidium iodide (PI) was then added to the AF cells. All the stained cells were analyzed using a flow cytometry platform for Annexin V-FITC and PI.

### 2.9. Suppression of Apoptosis by Z-LEHS-FMK

Z-LEHD-FMK (Caspase 9 inhibitor) was purchased from MedChemExpress (MCE), and the Cat. No. is HY-P1010.AF cells were seeded in 12-well culture plates at a density of 5 × 10^5^ cell/plate. All the plates were divided into four groups, with three plates each group. When the confluences of cells reached 80-90%, cell culture media were changed. The cells in one group were pretreated with 6 *μ*l Z-LEHD-FMK for 2 h after being infected with *Smad7* overexpression adenovirus. The other three groups were infected with *Smad7* overexpression adenovirus, empty adenovirus, or nothing. Cells were collected after 48 h to test apoptosis rate.

### 2.10. Immunofluorescence Analysis

AF cells were seeded on sheet glasses in plates. AF cells were fixed by 4% paraformaldehyde for 15 min, permeabilized with 0.1% Triton X-100 for 15 min, and then blocked by 5% BSA (bovine serum albumin) for 1 h. The AF cells were incubated with p-*Smad3* (rabbit monoclone antibody, 1 : 200, Abcam) and p-*Smad2* (rabbit monoclone antibody, 1 : 400, CST) primary antibodies overnight at 4°C. AF cells were washed with PBST thrice. Finally, AF cells were incubated with Cy3-conjugated AffiniPure goat anti-rabbit IgG (H+L). Photographs were taken by Olympus BX51.

### 2.11. Statistical Analysis

All data were analyzed using SPSS11.5 statistical software. Comparisons of data between groups were analyzed using one-way ANOVA, Kruskal-Wallis *H*-test, or independent-samples *t*-test according to the distribution of data and homogeneity of variance. *p* values less than 0.05 were considered statistically significant.

## 3. Results

### 3.1. *Smad7* Is Highly Expressed in the Human Degenerative Discs

**Figure 1:**
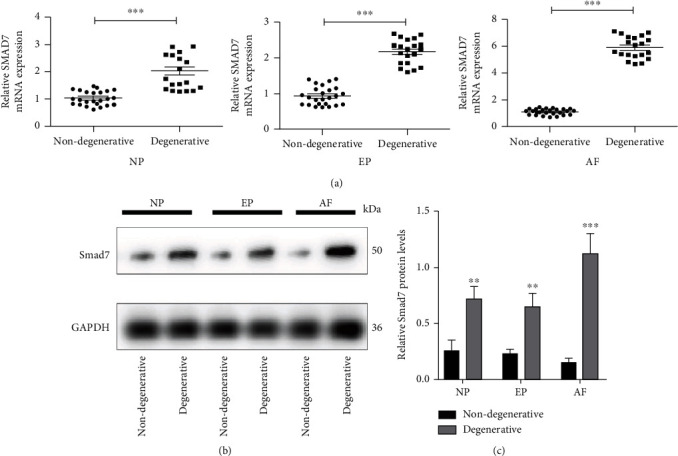
The expression of *Smad7* in human degenerative and non-degenerative intervertebral discs. (a) qRT-PCR assays to detect the mRNA expression of *SMAD7* in human degenerative and nondegenerative intervertebral discs. (b) Western blotting assays to detect the protein expression of *SMAD7* in human degenerative and nondegenerative intervertebral discs. *p* values less than 0.05 were considered statistically significant. NP: nucleus pulposus; AF: annulus fibrosus; EP: endplate; ^∗∗∗^*p* < 0.001,  ^∗∗^*p* < 0.01, and^∗^*p* < 0.05. All experiments were performed at least three times.

The expression of *Smad7* in degenerative NP, EP, and AF was significantly higher than that in the nondegenerative counterpart (Figure 1(a)). The *Smad7* protein expressions showed similar variation trends as compared with PCR analysis (Figure 1(b)). But *Smad7* expressions were much higher in the AF than in NP and EP, which indicated that *Smad7* might have a more important role in the AF than in the other components of the disc.

### 3.2. *IL-1β* Induces Apoptosis in Rat AF Cells through Its Regulations on *Smad7*

**Figure 2:**
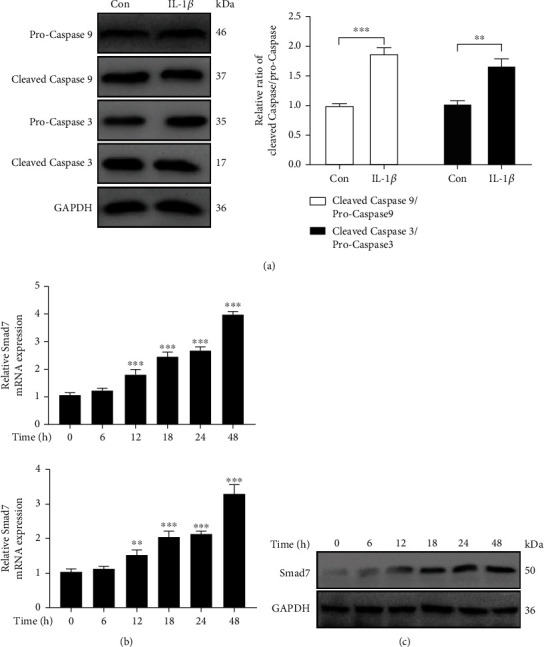
*IL-1β* increases the expression of *Smad7* in rat AF cells along with apoptosis. (a) Western blotting assays to detect the protein expression of cleaved-caspase 9 and cleaved-caspase 3 in AF cells treated with *IL-1β*. (b) qRT-PCR assays to detect the mRNA expression of *Smad7* in AF cells treated with *IL-1β* for various times (0, 6, 12, 18, 24, and 48 h). (c) Western blotting assays to detect the protein expression of *Smad7* in AF cells treated with *IL-1β* for various times (0, 6, 12, 18, 24, and 48 h). *p* values less than 0.05 were considered statistically significant. ^∗∗∗^*p* < 0.001,  ^∗∗^*p* < 0.01, and^∗^*p* < 0.05. All experiments were performed at least three times.

It has been proved that *IL-1β* induces apoptosis in AF cells [18, 24, 25]. We investigated whether *Smad7* was involved in the *IL-1β-induced apoptosis* of AF cells. With the stimulation of *IL-1β*, the apoptosis-related proteins, such as cleaved caspase-9 and cleaved caspase-3, significantly increased in the AF cells (Figure 2(a)). *Smad7* mRNA (Figure 2(b)) and protein (Figure 2(c)) expressions were also upregulated with time in these AF cells.

**Figure 3:**
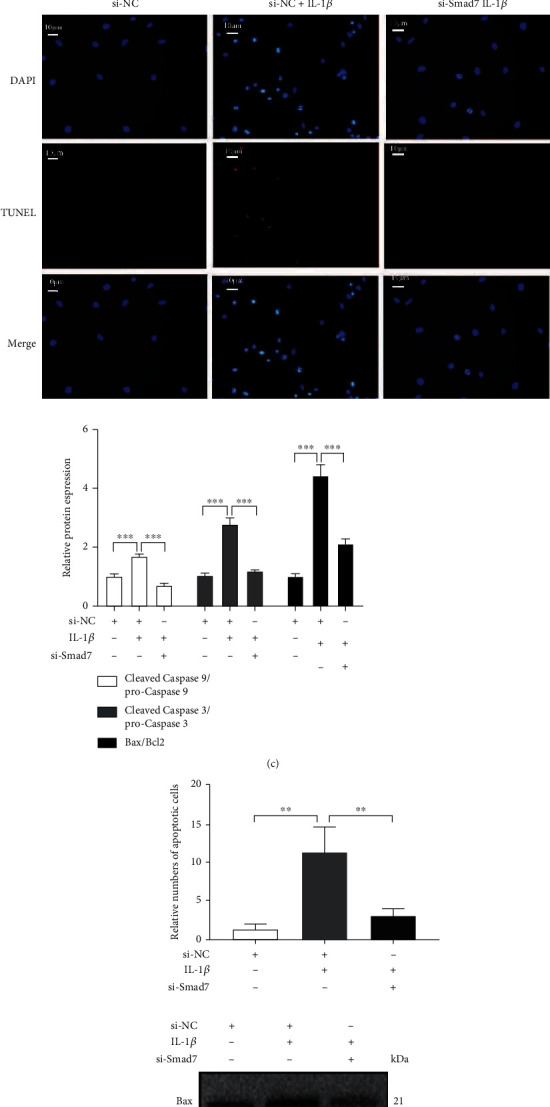
*Smad7* Knockdown alleviates the *IL-1β-induced apoptosis* of AF cells. (a) qRT-PCR assays to detect the mRNA expression of *Smad7* in AF cells treated with si-NC, si-*Smad7-1*, si-*Smad7-2*, and si-*Smad7-3*. (b) Western blotting assays to detect the protein expression of *Smad7* in AF cells treated with si-NC, si-Smad7-1, si-Smad7-2, and si-Smad7-3. (c) Representative pictures of TUNEL-stained AF cells treated with si-NC, si-NC+*IL-1β*, and si-*Smad7*+*IL-1β*. (d) Western blotting assays to detect the protein expression of Bax, Bcl-2, cleaved-caspase 9, and cleaved-caspase 3 in AF cells treated with si-NC, *IL-1β*, and si-*Smad7*+*IL-1β*. *p* values less than 0.05 were considered statistically significant. ^∗∗∗^*p* < 0.001,  ^∗∗^*p* < 0.01, and^∗^*p* < 0.05. All experiments were performed at least three times. Scale bar: 10 *μ*M.

Successful knockdown of *Smad7* in AF cells was verified at both the gene (Figure 3(a)) and protein (Figure 3(b)) levels. With the knockdown of *Smad7* in AF cells, the *IL-1β-induced apoptosis* was greatly inhibited (Figure 3(c)). The expression of apoptosis-related proteins, such as Bax/Bcl-2, cleaved caspase-9, and cleaved caspase-3, was also significantly suppressed (Figure 3(d)).

**Figure 4:**
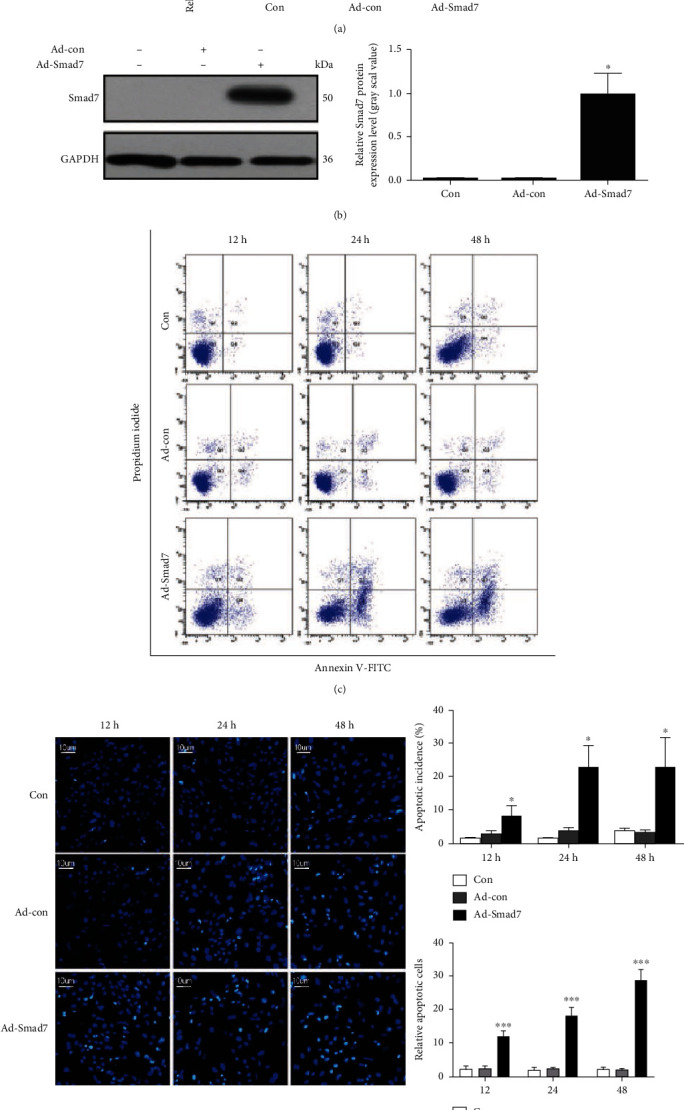
Overexpression of *Smad7* exaggerates the *IL-1β*-induced apoptosis (a) qRT-PCR assays to detect the mRNA expression of *Smad7* in AF cells treated with Con, Ad-Con and Ad-*Smad7*. (b) Western blotting assays to detect the protein expression of *Smad7* in AF cells treated with Con, Ad-Con, and Ad-Smad7. (c) Representative flow cytometry graphs of rat AF cells stained with Annexin V-FITC and PI. Gates were set as follows: Q1 (Annexin V^−^PI^+^), necrosis cells; Q2 (Annexin V^+^PI^+^), late-stage apoptotic cells; Q3 (Annexin V^−^PI^−^), live cells; Q4 (Annexin V^+^PI^−^), early-stage apoptotic cells. Apoptotic rates of three groups of rat AF cells at different time points. The percentages of Annexin V^+^ cells were calculated at Con, Ad-Con, and Ad-*Smad7* at 12, 24, and 48 h, respectively. (d) Rat AF cells stained by Hoechst 33285 at 12, 24, and 48 h. Original magnification: ×200. *p* values less than 0.05 were considered statistically significant. ^∗∗∗^*p* < 0.001,  ^∗∗^*p* < 0.01, and^∗^*p* < 0.05. All experiments were performed at least three times. Con: control rat AF cells; Ad-Con: rat AF cells infected by adenovirus taking empty plasmid; Ad-*Smad7*: rat AF cells infected by adenovirus taking *Smad7* overexpression plasmid. Original magnification: ×100. Scale bar: 10 *μ*M.

Overexpression of *Smad7* in AF cells with adenovirus infection was also successful (Figures 4(a) and 4(b)). On the contrary, with the overexpression of *Smad7* in AF cells, the *IL-1β-induced apoptosis* was greatly exaggerated (Figure 4(c)). More chromosome condensation and nuclear fragmentation under Hoechst 33258 staining were seen in the cells (Figure 4(d)). Taken together, these results indicated that *IL-1β-induced apoptosis* in rat AF cells through its regulations on *Smad7.*

### 3.3. *Smad7* Participates in IL-1*β*-Induced Apoptosis of Rat AF Cells via the Mitochondria Pathway

There are two main apoptotic pathways: the extrinsic (also called death receptor) pathway and the intrinsic (also called mitochondrial) pathway [26]. The extrinsic pathway requires external stimulation, and this occurs via a death receptor (DR). The intrinsic pathway involves a wide array of stimuli that are sensed intracellularly, including cytokine deprivation, DNA damage, and endoplasmic reticulum (ER) stress [27]. These diverse apoptotic stresses converge to trigger one crucial event—mitochondrial outer membrane permeabilization (MOMP) [28]. It is reported that *TGF-β1-*mediated apoptosis was associated with *Smad7*-dependent mitochondrial Bcl-2 expression [29] and *Smad7* increased *TGF-β*-mediated apoptosis in Mv1Lu cells [30]. These studies indicated that *Smad7* might lead to cell apoptosis via the mitochondria signaling pathway.

**Figure 5:**
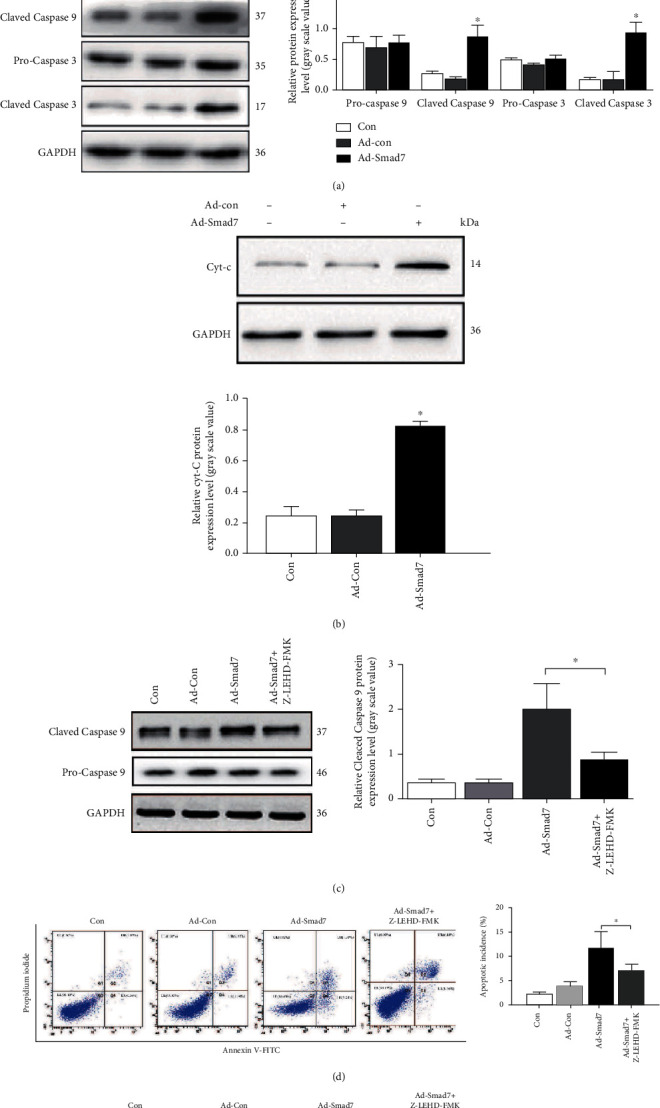
*Smad7* participates in IL-1*β*-induced apoptosis of rat AF cells via the mitochondria pathway. (a) Western-blot analysis of Bax, Bcl-2, Bcl-XL, procaspase 9, cleaved-caspase 9, procaspase 3, and cleaved caspase3 in whole cell lysis from Con, Ad-Con and Ad-*Smad7*-infected rat AF cells. (b) Western-blot analysis of Cyt-c in whole cell lysis from Con, Ad-Con, and Ad-Smad7 infected rat AF cells. (c) Western blot analysis of procaspase 9 and active cleaved-caspase 9. (d) Representative flow cytometry graphs of rat AF cells stained with Annexin V-FITC and PI after treatment with Z-LEHS-FMK. (e) Immunofluorescence assays of cleaved-caspase 9 in AF cells. ^∗∗∗^*p* < 0.001,  ^∗∗^*p* < 0.01, and^∗^*p* < 0.05. All experiments were performed at least three times. Con, Ad-Con, Ad-*Smad7*-infected rat AF cells, and Ad-*Smad7*-infected rat AF cells treated with Z-LEHS-FMK. Scale bar: 20 *μ*M.

To investigate whether *Smad7* participates in *IL-1β-induced apoptosis* of rat AF cells via the mitochondria signaling pathway, we measured the protein expression of different biomarkers of the mitochondria-mediated apoptosis. Western blot analysis revealed that the protein level of *Bax* in rat AF cells overexpressing *Smad7* significantly increased, while the levels of both *Bcl-2* and *Bcl-XL* significantly decreased, which resulted in even greater increase in the ratio of *Bax/Bcl-2* (Figure 5(a)). Meanwhile, the expression of both active cleaved caspase-3 and cleaved caspase-9 also increased significantly in the AF cells with *Smad7* overexpression. However, the protein expressions of procaspase-3 and procaspase-9 did not show any significant changes (Figure 5(a)). In addition, the expression of Cyt-c also increased significantly (Figure 5(b)). These results indicated that the mitochondrial pathway is likely involved in the *Smad7*-mediated apoptosis induced by *IL-1β*. *Smad7* may promote *Bax* expression by inhibiting *Bcl-2* and *Bcl-XL* expression and activating the mitochondria-mediated apoptosis pathway.

To further confirm the involvement of the mitochondria pathway in the *Smad7*-mediated apoptosis, AF cells with overexpression of *Smad7* were treated with Z-LEHD-FMK, an inhibitor of caspase-9. After treatment with Z-LEHD-FMK, the protein expression of active cleaved-caspase-9 in Ad-*Smad7*-infected cells was downregulated by one-fold (Figure 5(c)), which is consistent with results reported in previous studies [31–33]. The apoptosis percentage was significantly lower in Ad-*Smad7*-infected cells treated with Z-LEHD-FMK than in cells without Z-LEHD-FMK treatment; however, it was still higher than that in Con and Ad-Con cells (Figure 5(c)). This suggested that Z-LEHD-FMK could not completely inhibit the *Smad7*-mediated apoptosis in AF cells. Immunofluorescence assay for the detection of protein expression of cleaved caspase-9 showed that Ad-*Smad7* increased the level of cleaved caspase-9, while Z-LEHD-FMK weakened this effect of Ad-*Smad7* (Figure 5(d)). These results further provided evidence that *Smad7* participates in *IL-1β-induced apoptosis* of rat AF cells via the mitochondria signaling pathway.

### 3.4. *Smad7* Regulates the *IL-1β*-Induced Apoptosis in AF Cells by Interfering with the Formation of Smad2/Smad3/Smad4 Complex

**Figure 6:**
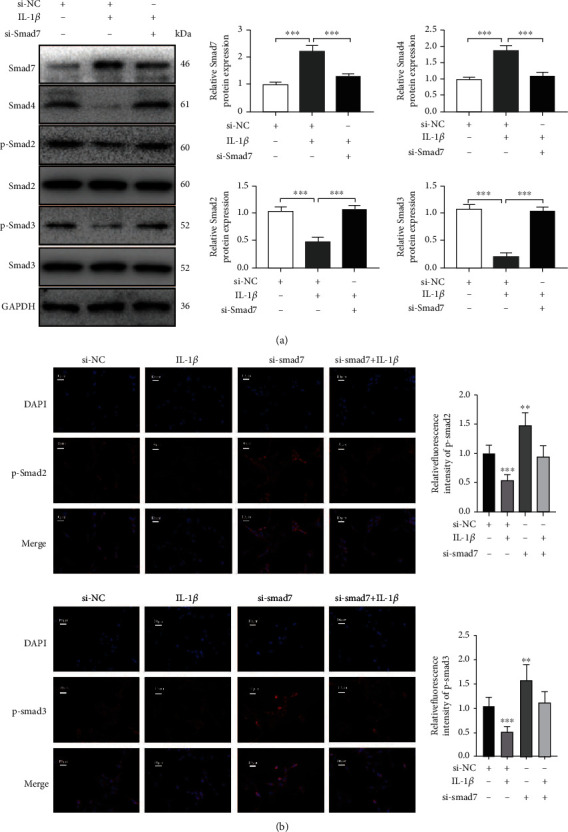
*Smad7* regulates the *IL-1β*-induced apoptosis in AF cells by interfering with the formation of Smad2/Smad3/Smad4 complex. (a) Western blotting assays to detect the protein expression of *Smad7*, *Smad4*, *p-Smad2*, *Smad2*, *p-Smad3*, and *Smad3* in AF cells treated with si-NC, *IL-1β*, and si-*Smad7+IL-1β*. (b) Immunofluorescence assays of *p-Smad2* and *p-Smad3* in AF cells treated with si-NC, *IL-1β*, *si-Smad7*, and *si-Smad7+IL-1β*. *p* values less than 0.05 were considered statistically significant. ^∗∗∗^*p* < 0.001,  ^∗∗^*p* < 0.01, and^∗^*p* < 0.05. All experiments were performed at least three times. Scale bar: 10 *μ*M.

With the stimulation of *IL-1β*, the protein expressions of *p-Smad2*, *p-Smad3*, and *Smad4* significantly decreased in the AF cells (Figure 6(a)). However, in cells with a successful *Smad7* knockdown, the expression of *p-Smad2*, *p-Smad3*, and *Smad4* was not affected by *IL-1β* treatment. Furthermore, in the cells with *Smad7* knockdown, the nuclear translocation of *p-Smad2*/*p-Smad3* significantly increased (Figure 6(b)). These results demonstrated that *Smad7* could regulate the *IL-1β-induced apoptosis* in AF cells by interfering with the formation of *Smad2/Smad3/Smad4* complex.

## 4. Discussion

*Smad7* is a negative regulatory protein involved in the signal transduction of *TGF-β* pathway. It blocks signal transduction and inhibits the transcription of *TGF-β* gene. In our previous study, we observed that *Smad7* was highly expressed in degenerative intervertebral discs of rats and that this was closely related to the catabolism and apoptosis of disc cells [13]. However, the underlying mechanisms remained unknown.

A clue indicating a potential role of *Smad7* in IVDD came from the studies showing that *TGF-β1* could promote proteoglycan synthesis and cell proliferation of NP and AF cells cultured *in vitro* and *in vivo* [7, 34–37]. As a downstream molecule of *TGF-β1*, exploring the role and mechanism of *Smad7* in intervertebral disc degeneration has grabbed our interest. In this study, we found that *Smad7* was highly expressed in human degenerative discs. Combined with the finding in our previous animal study that the expression of *Smad7* significantly increased in the degenerative discs of rats, we verified that *Smad7* should be involved in the pathophysiological process of intervertebral disc degeneration.

It has been proved that *IL-1β* has important roles in the pathogenesis of disc degeneration by stimulating the apoptosis in disc cells [17, 18]. In this study, we verified that *IL-1β* could stimulate apoptosis in the AF cells isolated and cultured from rats and found increased *Smad7* expression in the cells that along with this *IL-1β-induced apoptosis*. In order to investigate the role of *Smad7* in the apoptosis of AF cells and the underlying signaling pathway, *Smad7* was overexpressed or knocked down in the AF cells under *IL-1β* stimulation. Overexpression of *Smad7* in the AF cells exaggerated the *IL-1β-induced apoptosis*, while knockdown of *Smad7* in the cells significantly diminished the *IL-1β-induced apoptosis*. Meanwhile, we found that with the increase of apoptosis in the cells with *Smad7* overexpression, the proteins in the mitochondrial mediated apoptosis pathway, such as active cleaved caspase-3 and cleaved caspase-9, the ratio of Bax/Bcl-2, and Cyt-c increased significantly. However, the inhibitor of caspase-9, Z-LEHD-FMK, significantly diminished the apoptosis in these cells. *Smad7* regulated the apoptosis of the AF cells through the mitochondrial signaling pathway. Our results also demonstrated that Z-LEHD-FMK did not completely block the activation of caspase-9 in the AF cells; it did not completely offset the increased apoptosis in the AF cells with *Smad7* overexpression. The reasons for this incompetency of Z-LEHD-FMK might be as follows: (1) Z-LEHD-FMK did not completely bind to caspase-9 in the AF cells, resulting in an incomplete inhibition of apoptosis in the cells with *Smad7*-overexpression. (2) Caspase-9 in the middle of the apoptotic cascade is the upstream molecule of caspases 3, 6, and 7 [28, 38, 39]*. Smad7* activated the downstream molecules of caspase-9 through other means. (3) *Smad7* did not completely activate the apoptosis through the mitochondrial pathway, and hence, other pathways might be involved.

High expression of *Smad7* was reported in chondrocytes under *IL-1β* stimulation [19]. Although *IL-1β* is proved to be able to induce disc cell apoptosis and disc degeneration, the expression of *Smad7* in the AF cells under *IL-1β* stimulation has never been described. In our study, we found that *IL-1β* led to an increase in *Smad7* expression and a decrease in *p-Smad2*, *p-Smad3*, and *Smad4* expressions in the AF cells. After successful *Smad7* knockdown in the AF cells, stimulation of *IL-1β* did not change the expressions of *p-Smad2*, *p-Smad3*, and *Smad4*. Combined with previous data and studies [25, 40, 41], we believed that *Smad7* should be involved in the apoptosis of AF cells by interfering with the formation of the Smad2/Smad3/Smad4 complex and regulate the apoptosis through the mitochondrial signaling pathway.

In conclusion, our study demonstrated that *Smad7* was highly expressed in human degenerative intervertebral discs and that *Smad7* regulated apoptosis of the AF cells mainly through the mitochondria signaling pathway. *Smad7* might be a potential target for the prevention and treatment of IVDD.

## Data Availability

Data can be obtained from the corresponding authors if required.
